# Local innovations and country ownership for sustainable development

**DOI:** 10.2471/BLT.15.164483

**Published:** 2015-11-01

**Authors:** Taye Balcha, Haileyesus Getahun, Kestebirhan Admasu

**Affiliations:** a Ministry of Health, Addis Ababa, PO Box 1234, Ethiopia.; b World Health Organization, Geneva, Switzerland.

During the past two decades, access to primary health care has been dramatically improved in Ethiopia as a result of a national health extension programme. More than 40 000 community health extension workers have been trained to deliver preventive and basic curative interventions in villages across the nation.^1^ The health extension workers are trained for a year, then employed by the government to deliver 16 interventions targeted at maternal and child health, tuberculosis, human immunodeficiency virus (HIV), malaria, sexually transmitted infections, sanitation and hygiene at household level and in the community.

Recently, an organized community movement led by women – the health development army – has increased community engagement in health. The movement is led by more than 3 million women who identify local health challenges of critical importance and develop innovative solutions to address them. Examples of these local solutions include: purchasing ambulances for medical referrals; constructing short-term accommodation within rural health centres where pregnant mothers from remote areas can stay during the last days of their pregnancy; draining wetlands to reduce mosquito breeding places for malaria prevention.

The health extension programme has been associated with impressive health gains in Ethiopia. Life expectancy at birth has increased from 46.7 years in 1990 to 64 years in 2013. Between 1990 and 2010, the years of life lost due to premature mortality from measles and diarrhoeal diseases decreased by 86% and 52%, respectively. As a consequence, Ethiopia has achieved the target of reducing child mortality by two-thirds between 1990 and 2015.^2^ It has also met the targets for reducing the burden of malaria, HIV and tuberculosis through prevention and treatment interventions. Between 1990 and 2014, new HIV infections have fallen by 90%, malaria-related mortality by 75% and mortality from tuberculosis by 64%.^3^^–^^6^ The maternal mortality ratio has declined by 69%, slightly short of the target of 75%.^7^

Ethiopia has shared these experiences with other African countries by hosting field visits from Ghana, Kenya, Liberia, Namibia, Niger, Nigeria, Sierra Leone, South Sudan and Uganda. Technical advisers from the Ethiopian Ministry of Health have helped Namibia to replicate the experience of Ethiopia. Ethiopia is establishing an International Primary Health Care Institute, in close collaboration with international partners. The institute will promote south–south collaboration through training and research, with an emphasis on community engagement.

Consolidating these achievements, guided by a vision of becoming a lower middle-income country by 2025, Ethiopia is now planning for the attainment of the sustainable development goals. New national health policy and health sector development plans are intended to achieve health-related targets comparable to the best-performing middle-income countries by 2035. Ethiopia will deliver quality health care to all, irrespective of age, gender, socioeconomic status or place of residence. To ensure universal health coverage, community-based health insurance and a new social health insurance scheme are being introduced. Community-based health insurance covers people in rural communities who rely on subsistence livelihoods, while social health insurance will cover people employed in the formal sector.

Over the last two decades, a range of simple innovations have transformed global health. As part of its preparedness to meet the sustainable development goals, Ethiopia is establishing a mechanism to review locally-developed measures, stimulate new innovations and test and adopt innovations, tools and technological solutions that have proven effective elsewhere. These efforts will focus on key areas including neonatal health and early child development, maternal health, health services tailored to pastoralist communities, local evidence generation and translation into sound policy and practice. Monitoring and evaluation will be used to assess the impacts.

Innovations that respond to the local context require support, understanding and acceptance by partner organizations and international donors. Policies for attaining the sustainable development goals should emphasize country ownership, promotion of local innovations and independence from outside donors. After all, health is a basic human right that the government has a responsibility to support.
